# Beers-Fick criteria and drugs available through the Farmácia Dose Certa program

**DOI:** 10.1590/S1516-31802011000100004

**Published:** 2011-01-06

**Authors:** Giancarlo Lucchetti, Alessandra Lamas Granero Lucchetti, Sueli Luciano Pires, Milton Luiz Gorzoni

**Affiliations:** I MD. Geriatrics specialist, Geriatrics and Gerontology Sector, Santa Casa de São Paulo, São Paulo, Brazil.; II MD. Geriatrics specialist, Interdisciplinary Center for Aging Research and Care, Belo Horizonte, Minas Gerais, Brazil.; III MD, MSc. Professor of Geriatrics and Director of Hospital Dom Pedro II, Geriatrics and Gerontology Sector, Santa Casa de São Paulo, São Paulo, Brazil.; IV MD, PhD. Professor and head of Geriatrics and Gerontology Sector, Santa Casa de São Paulo, São Paulo, Brazil.

**Keywords:** Aged, Pharmaceutical preparations, Drug prescriptions, Iatrogenic disease, Adverse effects, Idoso, Preparações farmacêuticas, Prescrições de medicamentos, Doença iatrogênica, Reações adversas a medicamentos

## Abstract

**CONTEXT AND OBJECTIVE:**

Farmácia Dose Certa is a program available in the State of São Paulo that is a national reference for providing drugs free of charge to the population. Elderly people receiving care deserve special attention regarding drugs that are appropriate for their age group. The objective was to assess the drugs in the program considered to be inappropriate for the elderly.

**DESIGN AND SETTING:**

Descriptive study evaluating free drug distribution in the State of São Paulo, Brazil.

**METHODS:**

Following the criteria proposed by Beers and Fick (drugs or drug classes that should be avoided among elderly people, independent of the diagnosis or clinical condition, because of the high risk of side effects and because other, safer drugs are available), the drugs in the Farmácia Dose Certa program that might be inappropriate for elderly people and the levels of evidence for each drug included were assessed.

**RESULTS:**

Among the available drugs, 10 (25.6%) were included within the Beers-Fick criteria. The drugs selected were: amitriptyline, cimetidine, diazepam, digoxin, fluoxetine, methyldopa, nifedipine, promethazine, thioridazine and ferrous sulfate.

**CONCLUSION:**

The list of drugs available within the Farmácia Dose Certa program may be considered appropriate for the general population, but not completely for the elderly population. Adjusting this list to the pharmacological aspects of aging will reduce the risks of drug interactions, falls, mental confusion and excessive sedation that result from drugs that are considered inappropriate for consumption by elderly people.

## RESUMO

**CONTEXTO E OBJETIVO:**

Programa Dose Certa é programa estadual para disponibilizar medicamentos de forma gratuita à população do Estado de São Paulo que é referência nacional no assunto. O segmento de idosos na população assistida merece atenção especial quanto aos fármacos adequados a este segmento etário. O objetivo foi avaliar as drogas no programa que são impróprias para idosos.

**TIPO DE ESTUDO E LOCAL:**

Estudo descritivo avaliando a distribuição de medicamentos no estado de São Paulo.

**MÉTODOS:**

Seguindo-se os critérios propostos por Beers e Fick (medicamentos ou classes deles que deveriam ser evitados em idosos, independentemente do diagnóstico ou da condição clínica, devido ao alto risco de efeitos colaterais e pela existência de outros fármacos mais seguros), avaliaram-se quais medicamentos do programa Dose Certa são impróprios para idosos e os níveis de evidência para cada medicamento incluído.

**RESULTADOS:**

Dos medicamentos disponíveis, 10 (25,6%) foram incluídos nos critérios de Beers-Fick: amitriptilina, cimetidina, diazepam, digoxina, fluoxetina, metildopa, nifedipina, prometazina, tiorodazida e sulfato ferroso.

**conclusão:**

A lista de fármacos disponível no Programa Dose Certa pode ser considerada apropriada para a população em geral, mas não totalmente para idosos. Adequá-los a aspectos farmacológicos do envelhecimento reduzirá riscos de interações medicamentosas, quedas, confusão mental e sedação excessiva decorrente de medicamentos considerados impróprios para o consumo de idosos.

## INTRODUCTION

Approximately 14.5 million Brazilians are elderly (8.6% of the total Brazilian population).^1^ Over the past four decades, there has been a twofold increase in the rate of aging in this country, and the segment of the population aged 60 years or more has grown fastest.^2^ Because elderly people suffer from more chronic and degenerative diseases, they usually consume more drugs and, consequently, can suffer more adverse effects than seen in the general population.^3^ This can be explained by the physiological changes relating to aging, such as body composition and renal and hepatic function, which alter the pharma-cokinetics and pharmacodynamics of various drugs.^4^

With the aim of defining which groups of medications are potentially inappropriate for the elderly, Beers^5^ established criteria for this. These were later upgraded by Fick et al.^6^ and bring together a list of drugs or drug classes that should be avoided among the elderly, regardless of the diagnosis or clinical condition, because of the high risk of adverse effects and/or the existence of safer drugs. Following this normative standard, we sought to determine what percentage of potentially inappropriate drugs available in the Farmácia Dose Certa program (a reference program for dispensation of medicines free of charge in the State of São Paulo) met the Beers-Fick criteria.

## OBJECTIVE

The objective was to assess the drugs in the program that are considered to be inappropriate for the elderly.

## METHODS

This study consisted of an analysis on which drugs available through the Farmácia Dose Certa program of the São Paulo State Health Department met the Beers-Fick criteria.

The drugs available within the Farmácia Dose Certa program were obtained from the following sites: http://portal.saude.sp.gov.br and http://www.furp.sp.gov.br/dose_certa/dose.asp. These drugs are available without charges for the entire population of São Paulo, Brazil.

The inclusion criteria used by the present study were based on the Beers criteria, as modified by Fick et al.^6^ in 2003. These criteria were initially developed by Beers^5^ in 1997, through a consensus involving prominent people in the fields of geriatrics, pharmacology and psychopharmacology who had done an extensive review of scientific studies available at that time. In 2003, Fick et al.^6^ reviewed and updated these criteria (**Table 1**).

**Table 1.** Criteria for potentially inappropriate medication use among older adults independent of diagnoses or conditions.^6^ Only drugs available in Brazil are displayed

Benzodiazepines	Amiodarone
Lorazepam > 3.0 mg/day	Digoxin > 0.125 mg/day
Alprazolam > 2.0 mg/day	(except in atrial arrhythmias)
Chlordiazepoxide	Disopyramide
Diazepam	Methyldopa
Clorazepate	Clonidine
Flurazepam	Nifedipine
Amitriptyline	Doxazosin
Fluoxetine (daily)	Dipyridamole
Barbiturates (except phenobarbital)	Ticlopidine
Thioridazine	Non-steroidal anti-inflammatory
Meperidine	*Indomethacin*
Anorexic drugs	*Naproxen*
Amphetamines	*Piroxicam*
Antihistamines	Muscle relaxants and antispasmodics
Chlorpheniramine	Carisoprodol
Diphenhydramine	Chlorzoxazone
Hydroxyzine	Cyclobenzaprine
Cyproheptadine	Orphenadrine
Tripelennamine	Oxybutynin
Dexchlorpheniramine	Hyoscyamine
Promethazine	Propantheline
Chlorpropamide	Belladonna alkaloids
Non-associated estrogens (oral)	Ketorolac
Thyroid extract	Ergot and cyclandelate
Methyltestosterone	Laxatives
Nitrofurantoin	Bisacodyl
Ferrous Sulfate	Cascara sagrada
Cimetidine	Mineral Oil

Since then, the Beers-Fick criteria have been used in population studies to analyze administrative data in intervention studies and regulation of drugs for nursing homes. They are also used to assess prescription patterns, medical education, clinical outcomes, costs and use of public health services.^6^ The criteria are divided as follows:^6^ (1) drugs or drug classes that should generally be avoided for people over 65 years of age, because they are ineffective or have a high risk or unnecessary adverse effects when a safer alternative is available; and (2) medications that should not be used for elderly patients with specific known conditions.

We analyzed all 65 drugs listed in the Farmácia Dose Certa program of the State of São Paulo. We decided to exclude 13 antibiotics from the analysis because they were not used on a regular basis; two drugs in the form of creams; five oral contraceptives because of their predominant use in younger age groups; a salt for oral rehydration used among children; and two vitamins. Drugs that had more than one presentation were considered as only one drug (there were three such drugs). For each drug available through the Farmácia Dose Certa program and included in the Beers-Fick criteria, we decided to show the level of evidence regarding higher number of adverse reactions in elderly individuals, compared with non-elderly individuals (**Table 2**).

**Table 2.** List of drugs provided by the Farmácia Dose Certa program in São Paulo, reasons for inappropriateness for the elderly according to the Beers-Fick criteria^6^ and levels of evidence for inclusion

Available drugs	Reason to be inappropriate	Level of Evidence	References
Aspirin 100 mg and 500 mg tablet			
Acid Valproic 250 mg tablet			
Aminophylline 100 mg tablet			
**Amitriptyline hydrochloride 25 mg tablet**	**Anticholinergic and sedative properties**	**1**	**14,15,16,17**
Biperiden 2 mg tablet			
Captopril 25 mg tablet			
Carbamazepine 200 mg tablet			
Lithium carbonate 300 mg tablet			
**Cimetidine 200 mg tablet**	**Central nervous system side effects including mental confusion**	**4**	**18, 19,20**
Clomipramine 25 mg caplet			
Clonazepam 2 mg tablet			
Chlorpromazine 25 mg tablet			
Chlorpromazine 100 mg tablet			
**Diazepam 10 mg tablet**	**Long half-life, prolonged sedation, increase risk of falls and fractures**	**2**	**21,22,23**
Diclofenac sodium 50 mg film-coated tablet			
Digoxin 0.25 mg tablet	Renal clearance dysfunction may lead to increase toxicity risks	2	24,25,26
Dipyrone 500 mg/ml drops			
Phenytoin 100 mg tablet			
Phenobarbital 100 mg tablet			
**Fluoxetine 20 mg tablets/caplets**	**Long half-life, can lead to overstimulation of the central nervous system, sleep disorders and excessive agitation**	**5**	6,27,28*,29*,30*
Furosemide 40 mg tablet			
Glibenclamide 5 mg tablet			
Haloperidol 5 mg tablet			
Hydrochlorothiazide 25 mg tablet			
Aluminum hydroxide 62 mg/ml oral suspension			
Imipramine 25 mg caplet			
**Methyldopa 250 mg film-coated tablet**	**May cause bradycardia and exacerbate depression in older patients**	**5**	31,32,33*,34*
Metoclopramide 10 mg tablet			
Naltrexone 50 mg tablet			
**Nifedipine 20 mg film-coated tablet**	**Potential for hypertension and constipation**	**2**	**35,36,37**
Nitrazepam 5 mg tablet			
Nortriptyline 25 mg tablet			
Paracetamol 200 mg/ml oral solution			
**Promethazine 25 mg tablet**	**Anticholinergic effects**	**3**	**38,39**
Propranolol 40 mg tablet			
Salbutamol 2 mg/5 ml syrup			
Sertraline 50 mg tablet			
**Ferrous sulfate drops**	**Doses above 325 mg/day may increase constipation**	**5**	**6**
**Thioridazine 100 mg tablet**	**Great potential for central nervous system and extrapyramidal effects**	**5**	**6**

Levels of Evidence: 1. Systematic review (SR) of randomized controlled trials (RCTs) or individual RCT; 2. Cohort studies (CS) or SR of CS or “outcome” research or ecological research; 3. Case-control studies(CCS) or SR of CCS; 4. Case series or poor-quality cohorts or poor-quality CCS; and 5. Expert opinion without explicit critical appraisal

* Studies showing no differences in adverse events, in comparison with other drugs.

The evidence for each drug was based on data from the Oxford Centre for Evidence-based Medicine (http://www.cebm.net). The levels of evidence were classified as follows: 1. systematic review (SR) of randomized controlled trials (RCTs) or an individual RCT; 2. cohort studies (CS) or SR of CS, or “outcomes” research or ecological research; 3. case-control studies (CCS) or SR of CCS; 4. case series or poor-quality cohorts or poor-quality CCS; and 5. expert opinion without explicit critical appraisal.

## RESULTS

Out of the 65 drugs, 26 were excluded because they fulfilled the exclusion criteria, as described in the Methods. The final list was composed of 39 drugs. From these, 10 (25.6%) were included within the Beers-Fick criteria, particularly the antihypertensive and psychotropic drugs (antipsychotics, benzodiazepines and antidepressants) (**Table 2**). The following drugs were included as potentially inappropriate: amitriptyline, cimetidine, diazepam, digoxin, fluoxetine, methyldopa, nifedipine, promethazine, thioridazine and ferrous sulfate. The level of evidence for each drug included is shown in **Table 2**. The percentages of drugs classified as appropriate or inappropriate according to the Beers-Fick criteria were divided into groups according to the recommendations of the National List of Essential Medications (Relação Nacional de Medicamentos Essenciais; Rename)^7^ and are summarized in **Table 3**.

**Table 3.** Appropriate and inappropriate drugs according to the Beers-Fick criteria,^6^ divided into groups according to the National List of Essential Medications (Relação Nacional de Medicamentos Essenciais)^8^ and available through the Farmácia Dose Certa program

Drug class or group	Appropriate	Inappropriate	Total	Percentage of inappropriate drugs
Antiallergics	-	1	1	100.0%
Antiulcer drugs	-	1	1	100.0%
Inotropes	-	1	1	100.0%
Antidepressants	2	2	4	50.0%
Antihypertensives	3	2	5	40.0%
Anti-anxiety drugs	2	1	3	33.3%
Anticonvulsants	4	-	4	0.0%
Hypoglycemic drugs	2	-	2	0.0%

## DISCUSSION

The Farmácia Dose Certa program is a national reference program that has been providing free drugs to the general population since 1995.^8^ According to the program website, by the year 2008, 14.5 billion drug doses had been exempted from charges for the São Paulo population since its beginning.

The drugs chosen for the program were selected because they were essential for use in primary healthcare. New drugs can be included or existing drugs can be excluded, within the scope of the program and according to the demand from primary healthcare units.

In 1995, when the program was first implemented, 7.2% of the population of Brazil was 60 years of age or over. Within this context, the Farmácia Dose Certa program was designed for younger age groups that, although still in the majority today, have been rapidly reducing in numbers and proportions in the Brazilian population.^1^ Thus, this program requires readjustment for this new group of elderly people, which needs different types of drugs.

Aging is associated with greater numbers of patients with chronic diseases. This, in turn, leads to increased drug consumption^9^ and increased frequency of significant adverse effects, drug interactions and polypharmacy.^10^ Elderly people have smaller volumes of water in their bodies, which leads to greater bioavailability of water-soluble drugs (for example, lithium and digoxin). However, lipid-soluble drugs like diazepam have a higher volume of distribution due to increased fat content in the body composition of this group. Other conditions that contribute towards uneven biodistribution of medicines among the elderly are the following: (1) plasma albumin concentration tends to be lower than in young adults, thereby lowering the binding capacity of drugs and resulting in increased plasma-free fractions and increased volume of distribution; and (2) progressive reduction in the capacity for renal excretion (due to the normal aging process and/or chronic diseases like hypertension and diabetes mellitus), thereby prolonging the half-life of drugs and increasing the likelihood of adverse effects.^11^

From this viewpoint, there is a need to evaluate which drugs are safe and effective and which should be avoided for elderly individuals. Criteria such as Beers-Fick^6^ seek to provide warnings regarding groups of drugs that are potentially inappropriate for this age group.

A percentage of the population, usually without the financial resources to afford regular consumption of drugs, relies on government support programs to gain access to medicines. There is thus a need to add flexibility to these programs, in order to monitor current and future demographic shifts.

From our evaluation of the Farmácia Dose Certa program in São Paulo, we found that approximately 25.0% of the drugs available were potentially inappropriate for elderly individuals according to the Beers-Fick criteria. Although neither the present study nor the original papers by Beers^5^ and Fick et al.^6^ had the aim of analyzing alternatives, we can make the observation that most of these drugs may be replaced with others that are more suitable for this age group.

This deserves attention, since certain medications that are usually prescribed for diseases that are common in old age, such as those with cardiovascular or central nervous system action, fulfill the criteria for inappropriateness according to Beers and Fick, yet form part of the program list.

This can be explained by the high prevalence of neurodegenerative diseases (such as Parkinson's disease and Alzheimer's disease) and mood disorders (i.e. depression and anxiety),^12^ which are associated with increased susceptibility to anticholinergic effects, i.e. sedation, falls or delirium.^13^

Some drugs included in the Beers-Fick criteria deserve additional comments. (1) amitriptyline presents anticholinergic effects and sedation, with level of evidence = 1;^6,14-17^ (2) cimetidine has central nervous system side effects, including mental confusion, with level of evidence = 4;^18-20^ (3) diazepam has a long half-life, produces sedation and predisposes towards falls and fractures, with level of evidence = 2;^6,21-23^ (4) digoxin gives rise to renal clearance dysfunction, which may lead to increase toxicity risks, with level of evidence = 2;^6,24-26^ (5) fluoxetine has a long half-life, which leads to a risk of central nervous system overstimulation, sleep disorders and agitation, with level of evidence = 5.^6,27^ However, some studies have not shown any differences in adverse events, when comparing fluoxetine with other drugs,^28-30^; (6) methyldopa, which presents bradycardia and depression in the elderly, with level of evidence = 5.^6,31,32^ However, some studies showed no differences in adverse events, when comparing methyldopa with other drugs;^33,34^ (7) nifedipine presents hypotension and constipation, with level of evidence = 2;^6,35-37^ (8) thioridazine is a typical neuroleptic with great potential for extrapyramidal and central nervous system symptoms, with level of evidence = 5;^6,38^ (9) promethazine has anticholinergic effects, with level of evidence = 3;^38,39^ and (10) ferrous sulfate, which at doses greater than 325 mg/day may increase constipation, with level of evidence = 5.^6^

Although most of these drugs may be replaced by more suitable ones for this age group, there are some classes of drugs with few options for physicians, such as antiulcer drugs and drugs for allergies. For example, antiulcer drugs (in this case, cimetidine) may be replaced by proton pump inhibitors (i.e. omeprazole, pantoprazole and lansoprazole, among others). Similarly, drugs for allergies (in this case promethazine) could be replaced by fexofenadine or loratadine. Drugs for treating diseases with high prevalence among the elderly that are often difficult to control, such as hypertension and depression, also have significant limitations regarding prescription, because of the high numbers of them that are deemed inappropriate according to these criteria.

Although not on the list of Beers-Fick criteria, other drugs such as biperiden (cognitive and balance problems),^40^ clonazepam (sleepiness and decreased reflexes),^41^ diclofenac sodium (gastrointestinal bleeding and decreased glomerular filtration rate),^42^ glibenclamide (prolonged hypoglycemia),^43^ nitrazepam (fall risk,^44^ disorientation, postural hypotension and cognitive dysfunction^45^), imipramine (orthostatic hypotension and falls^46^), haloperidol (tardive dyskinesia^47^) and lithium (prolonged half-life due to changes in renal function with higher risk of toxic levels^48^) are available on the list of the Farmácia Dose Certa program. In view of the effects that have been observed with the use of these drugs, they should be used with caution among this age group.

At no time did we question the scope and quality of the program, which has distributed more than 14 billion drug doses since its inception. Furthermore, the Farmácia Dose Certa program is supposed to cover most of the needs of the general population. However, if the program were better adapted to the pharmacological aspects of aging, the risks of drug interactions, falls, mental confusion and excessive sedation among elderly people would be reduced. Similarly, studies have shown that the use of potentially inappropriate medication increases healthcare spending^49^ and may lead to higher rates of hospitalization and mortality.^50^ Therefore, such medications should be replaced by safer ones.^51^

Certain limitations to the present study need to be mentioned. First, we used the Beers-Fick criteria. Although these are among the most widely used criteria for research, they may not represent or agree with the opinions of some specialists. In order to minimize this bias and help prescribing physicians, we decided to include the levels of evidence for each drug included. Second, the use of these criteria to assess the distribution of free drugs has rarely been addressed in the medical literature. This raises new questions, such as whether there would be good results in interventional studies, when comparing outcomes before and after these changes.

According to Lau et al.,^50^ studies on the subject are needed in order to expand the acceptability of these criteria among public health experts, facilitate medical education and prescriptions for the elderly, and even to facilitate drug regulation.

## CONCLUSION

In summary, this study points towards the need to revise the Farmácia Dose Certa program, in order to cover older consumers. This can be done through the introduction of new drugs or through a new list that is specific for the population over 60 years of age.
